# An Evolutionary Analysis of the *Secoviridae* Family of Viruses

**DOI:** 10.1371/journal.pone.0106305

**Published:** 2014-09-02

**Authors:** Jeremy R. Thompson, Nitin Kamath, Keith L. Perry

**Affiliations:** 1 Department of Plant Pathology and Plant-Microbe Biology, Cornell University, Ithaca, New York, United States of America; 2 Laboratory of Malaria and Vector Research, National Institute of Allergy and Infectious Diseases, Bethesda, Maryland, United States of America; Institute of Infectious Disease and Molecular Medicine, South Africa

## Abstract

The plant-infecting *Secoviridae* family of viruses forms part of the *Picornavirales* order, an important group of non-enveloped viruses that infect vertebrates, arthropods, plants and algae. The impact of the secovirids on cultivated crops is significant, infecting a wide range of plants from grapevine to rice. The overwhelming majority are transmitted by ecdysozoan vectors such as nematodes, beetles and aphids. In this study, we have applied a variety of computational methods to examine the evolutionary traits of these viruses. Strong purifying selection pressures were calculated for the coat protein (CP) sequences of nine species, although for two species evidence of both codon specific and episodic diversifying selection were found. By using Bayesian phylogenetic reconstruction methods CP nucleotide substitution rates for four species were estimated to range from between 9.29×10^−3^ to 2.74×10^−3^ (subs/site/year), values which are comparable with the short-term estimates of other related plant- and animal-infecting virus species. From these data, we were able to construct a time-measured phylogeny of the subfamily *Comovirinae* that estimated divergence of ninety-four extant sequences occurred less than 1,000 years ago with present virus species diversifying between 50 and 250 years ago; a period coinciding with the intensification of agricultural practices in industrial societies. Although recombination (modularity) was limited to closely related taxa, significant and often unique similarities in the protein domains between secovirid and animal infecting picorna-like viruses, especially for the protease and coat protein, suggested a shared ancestry. We discuss our results in a wider context and find tentative evidence to indicate that some members of the *Secoviridae* might have their origins in insects, possibly colonizing plants in a number of founding events that have led to speciation. Such a scenario; virus infection between species of different taxonomic kingdoms, has significant implications for virus emergence.

## Introduction

The *Picornavirales* order contains viruses that infect a wide range of eukaryotic organisms including vertebrates (*Picornaviridae*), arthropods (*Dicistroviridae*), plants (*Secoviridae*), insects (*Iflaviridae*) and algae (*Marnaviridae*) [1]. Members of the *Picornaviridae* include rhinoviruses, poliovirus, *Foot-and-mouth disease virus*, and *Hepatitis A virus*. The plant-infecting members of the *Picornavirales* are in the *Secoviridae* family. Most secovirid species fall within the *Comovirinae* subfamily which contains the *Nepovirus*, *Comovirus* and *Fabavirus* genera [2]. However, in the last decade or so, a number of novel more distantly related viruses have been characterized; these include *Apple latent spherical virus* (ALSV)[3], *Cherry rasp leaf virus* (CRLV)[4], *Satsuma dwarf virus* (SDV)[5], *Strawberry mottle virus* (SMoV)[6], *Strawberry latent ringspot virus* (SLRSV)[7] and *Tomato torrado virus* (ToTV)[8]. The agronomic importance of members of the *Secoviridae* is significant: *Grapevine fanleaf virus* (GFLV) is the oldest and most widespread viral disease to affect grapevine, being first documented in 1865 [9], while rice tungro disease, caused by a combination of two viruses, one of which is the secovirid *Rice tungro spherical virus* (RTSV) emerged in the 1960s to seriously disrupt rice production in Asia [10]. More recently multiple members of the newly described *Torradovirus* genus show signs of emergence in tomatoes [11], [12]. Their present impact on a wide range of agronomically important crops combined with their continuing emergence means that understanding the *Secoviridae* from an evolutionary perspective will enhance our ability to develop adequate control strategies against present and future threats.

Members of the *Picornavirales* are all characterized as having a positive-sense single-stranded RNA genome with a conserved module incorporating a superfamily 3 helicase (HEL), a chymotrypsin-like protease (PRO) and RNA-dependent RNA polymerase (RdRp) functions. Large exons are proteolytic processed via post-translational cleavage into discrete structural and/or functional proteins. Virus particles are non-enveloped, icosahedrons of around 30 nm in diameter made up of 60 capsomers each containing three jelly-roll domains. Except for members of the *Secoviridae*, of which almost all are bipartite, viruses in the order *Picornavirales* have non-segmented genomes. In the vast majority of cases the viral RNA is polyadenylated at the 3’ terminus. A small virus-encoded protein (VPg), predicted for most of the species, has been shown to be covalently attached to the 5’ terminus [1]. The coat protein (CP) of the *Secoviridae* consists of either one, two or three cleaved peptides, depending on the genus or species [2], [13]. Upstream of the CP is the movement protein (MP) which is required for cell-to-cell movement, although its biological function has only been verified for a small number of viruses. There exists upstream of the HEL and some MP functional domains regions of low levels of conservation. Except for the *Nepovirus* and *Comovirus* genera the functions of these regions remain unknown [13].

Beyond a number of reports measuring selection pressures and detecting specific examples of recombination there is limited information on the evolution of the *Secoviridae*. High levels of purifying selection for the CP and MP have been calculated for GFLV strains isolated from California [14]. RTSV strains taken from endemic regions in South East Asia also exhibited the characteristics of purifying selection for the CP [15]. Equally, an analysis of 30 global isolates of *Broad bean wilt virus-2* (BBWV-2) found evidence of strong purifying selection exerted on four functional domains including the CP and MP [16]. Recombination in GFLV appears to have occurred both at an intraspecific level and with the closely related *Arabis mosaic virus* (ArMV), at an interspecific level [17]–[21]. This intimate relationship is believed to have spawned *Grapevine deformation virus* which appears to be a mosaic between GFLV and ArMV [22]. For the comovirus *Bean pod mottle virus* (BPMV) Zhang et al. [23] identified a naturally occurring partial diploid reassortant strain that contained recombinant sequences derived from different BPMV strains and which could be replicated by coinfection and passaging. Recombinants have also been experimentally generated between nepoviruses *Tobacco black ring virus* (TBRV) and *Grapevine chrome mosaic virus* (GCMV) [24]. Beyond the *Comovirinae* sequence analyses of certain functional domains have identified a chimeric-like composition to secovirid genomes that extends outside of the *Secoviridae*. The CPs of CRLV and ToTV are reported to have highest identities with non-plant infecting members of the *Picornavirales* [4], [25]. In this study, by using a comparative genomics approach we set out to identify the tendencies underlying the genetics and evolution of the entire *Secoviridae* family. The results obtained help define the *Secoviridae* as an emerging group of viruses that we postulate in some cases originated in insects.

## Materials and Methods

### Sequence data collection and preparation

**Figure 1:**
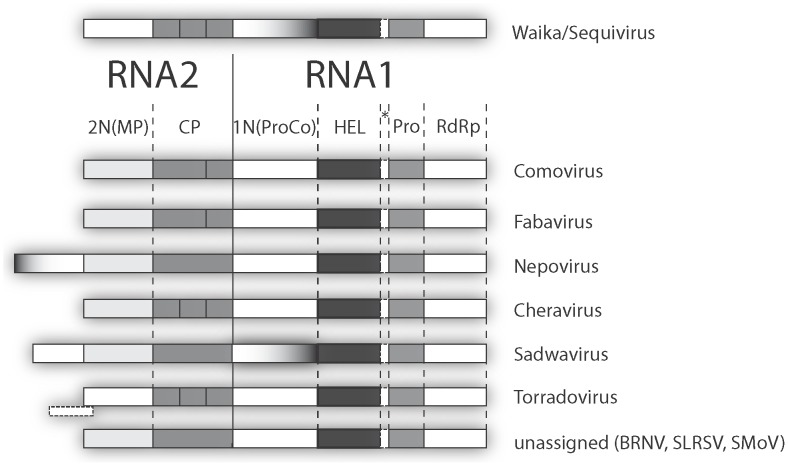
Simplified schematic of the genomic organization of the *Secoviridae*. The names of eight taxa are indicated on the right of the depicted genomes. The monopartite genomes of the waikaviruses and sequivirus are diagrammed at the top from left to right in a 5’ to 3’ direction. Only the protein-encoding regions are shown, delineated as shaded rectangles. The remaining taxa/genera have bipartite genomes. The functional domain order in the two RNAs is conserved and they are displayed with the RNA2 on the left, separated from the RNA1 by a solid vertical line. This arrangement allows the coding regions of the bipartite viruses to be aligned with the monopartite genomes. The functional regions from left to right are: the putative movement protein or 2N terminal protein 2N(MP), the coat protein (CP), the putative protease cofactor or 1N terminal protein 1N(ProCo), the helicase (HEL), the protease (Pro) and the RNA-dependent RNA polymerase (RdRp). Putative additional N-terminal genes are shown with dotted borders for the genera *Nepovirus* (of various sizes depending on subgroup), *Sadwavirus* and *Torradovirus*. The existence of a translation product or function for the latter virus has still not been demonstrated and was thus excluded from the analyses. * indicates the putative 5'-genome-linked protein (VPg). *Black raspberry necrosis virus* (BRNV), *Strawberry latent spherical virus* (SLRSV), *Strawberry mottle virus* (SMoV).

The full-length nucleotide sequences of 27 secovirids were downloaded from the NCBI GenBank database (http://www.ncbi.nlm.nih.gov) (Table 1). Selection was based on those species that were described as full-length and were sufficiently annotated to allow for the majority of genes to be identified and delineated as discrete sequences. This therefore precluded the initial selection of partial or insufficiently annotated sequences (eg. *Raspberry ringspot virus*). Full-length sequences were analyzed in Vector NTI (Invitrogen) with each open reading frame separated into its putative functional domains - Protease co-factor (ProCo), Helicase (HEL), Viral protein genome-linked (VPg), Protease (PRO), RNA-dependent RNA-polymerase (RdRp), Movement protein (MP) and Coat protein (CP) - as determined by reported cleavage sites, determined either experimentally or *in silico*. Since for some species there was functional ambiguity and/or additionally reported in-frame domains at the N-termini of ProCo and MP sequences, designation as the 1N(ProCo) and 2N(MP) domains, respectively, was more appropriate and adopted throughout this study (Fig.1). Because of its unusual position and unknown function, the frame-shifted 5’ domain identified in torradoviruses was not considered for analysis [12]. Individual CP sequences were selected from the Genbank database on the basis of whether a definitive year of isolation could be associated either by means of the annotation or from a cited publication.

**Table 1:** List of Secoviridae isolates and related sequences used in the analyses, including their acronyms and Genbank accession numbers.

Genome	Subfamily	Genus	Species	Acronym	RNA 1	RNA 2
bipartite	Comovirinae	*Comovirus*	*Bean pod mottle virus*	BPMV	NC_003496.1	NC_003495.1
			*Cowpea mosaic virus*	CPMV	NC_003549.1	NC_003550.1
			*Cowpea severe mosaic virus*	CPSMV	NC_003545.1	NC_003544.1
			*Radish mosaic virus*	RaMV	NC_010709.1	NC_010710.1
			*Red clover mottle virus*	RCMV	NC_003741.1	NC_003738.1
			*Squash mosaic virus*	SqMV	NC_003799.1	NC_003800.1
		*Fabavirus*	*Broad bean wilt virus-1*	BBWV1	NC_005289.1	NC_005290.1
			*Broad bean wilt virus-2*	BBWV2	NC_003003.1	NC_003004.1
		*Nepovirus*	*Arabis mosaic virus*	ArMV	AY303786.1	NC_006056.1
			*Beet ringspot virus*	BRSV	NC_003693.1	NC_003694.1
			*Blackcurrant reversion virus*	BRV	NC_003509.1	NC_003502.1
			*Cycas necrotic stunt virus*	CNSV	NC_003791.1	NC_003792.2
			*Grapevine chrome mosaic*	GCMV	NC_003622.1	NC_003621.1
			*Grapevine fanleaf virus*	GFLV	NC_003615.1	NC_003623.1
			*Tomato ringspot virus*	ToRSV	NC_003840.1	NC_003839.2
			*Tobacco ringspot virus*	TRSV	NC_005097.1	NC_005096.1
		*Cheravirus*	*Apple latent spherical*	ALSV	NC_003787.1	NC_003788.1
			*Cherry rasp leaf virus*	CRLV	AY764390.2	AY122330.2
		*Sadwavirus*	*Satsuma dwarf virus*	SDV	NC_003785.2	NC_003786.2
		*Torradovirus*	*Tomato marchitez virus*	ToMarV	NC_010987.1	NC_010988.1
			*Tomato torrado virus*	ToTV	DQ388879.1	DQ388880.1
		*unassigned*	*Strawberry latent ringspot virus*	SLRSV	NC_006964.1	NC_006965.1
			*Black raspberry necrosis virus*	BRNV	DQ344639.1	DQ344640.1
			*Strawberry mottle virus*	SMoV	NC_003445.1	NC_003446.1
monopartite		*Waikavirus*	*Maize chlorotic dwarf virus*	MCDV	NC_003626.1	
			*Rice tungro spherical virus*	RTSV	NC_001632.1	
		*Sequivirus*	*Parsnip yellow fleck virus*	PYFV	NC_003628.1	

### Alignments, selection pressures, nucleotide substitution rates

Nucleotide sequences of the seven identified functional domains – 1N(ProCo), HEL, VPg, PRO, RdRp, 2N(MP) and CP were aligned using the MUSCLE [26] codon align option in the MEGA4.0 suite [27]. Each alignment was screened for significant breakpoint signals using the GARD program [28] with standard default settings. Saturation of all alignments was assessed using DAMBE [29]. Global selection pressures were analyzed by determining the ratio of non-synonymous to synonymous (d_N_/d_S_) nucleotide substitutions using the Single-Likelihood Ancestor Counting (SLAC) method of the HYPHY program [30]. Evidence for episodic diversifying selection was screened using the branch-site REL method [31], at the datamonkey website (http://www.datamonkey.org/). Both SLAC and FUBAR [32] methods were used to identify codon specific selection. For SLAC and REL methods input neighbor-joining trees, generated within the programs themselves, were used with each alignment. For the former, a GTR substitution model – determined as the optimal for every alignment using JModeltest [33] - was used calculating 95% confidence intervals for a χ^2^ distribution. For the latter, alignments that generated trees with a single branch where episodic diversifying selection was identified were only considered positive if the same branches were identified during a re-run with maximum likelihood input parameters. Nucleotide substitution rate estimates were determined using BEAST 1.7 [34], [35]. The optimal substitution model for each alignment, identified using JModeltest [33], was used to compare against the simpler default HKY substitution model in the BEAUTi program in combination with different priors for the molecular clock (strict, lognormal relaxed, exponential relaxed) and tree (Bayesian skyline, exponential and constant population) all with codon partitioning. All sequences were prior ‘tip-dated’ with their year of isolation reported in the NCBI GenBank submission. BEAST results were analyzed in the Tracer program, each alignment being compared pairwise to obtain Bayes Factors and the marginal likelihood for each prior combination; the analysis with the largest marginal likelihood being selected as the best (results available on request). Finally, to assess the temporal signal, the best analyses were then compared using an identical BEAST run but with the ‘tip-dates’ randomized. BEAST runs were continued until all relevant parameters converged – discarding 10% of MCMC chains as burn-in. A highest density probability (HPD) of 95% was used to assess statistical significance.

### Trees

Maximum clade credibility trees with statistical support in the form of Bayesian posterior probabilities at each node were generated using BEAST and processed in the programs TreeAnnotator and FigTree. For maximum likelihood (ML) trees the MUSCLE alignments of each of the seven identified functional domains – 1N(ProCo), HEL, VPg, PRO, RdRp, 2N(MP) and CP and their respective optimal substitution models, as determined by JModeltest, were analyzed in PAUP*[36] using a heuristic search with 100 bootstrap replicates.

### Detection of recombination

Evidence for recombination prior to calculating selection pressures was carried out, as mentioned above, using GARD [28]. The GARD algorithm initially works by looking for the number and placement of breakpoints that yields the best Akaike Information Criteria (AICc); a measure of the goodness of fit. However, any improvement in model fit could be due to a number of factors (eg. spatial rate variation, heterotachy) other than a change in the tree topology, which would be the primary signature for recombination. GARD subsequently checks for tree congruence either side of the putative breakpoint using the Kishino-Hasegawa (KH) test [37]. For calculating selection pressures therefore detection of significant incongruity in an alignment was adjusted prior to analysis. Evidence of putative local recombination events more specific to individual viruses was determined using the RDP3 software [38] in a stepwise fashion as previously described [39]. This involved realigning subsets of viral sequences that were determined monophyletic in maximum likelihood inferred trees, thereby reducing the amount of sequence noise and the possibility of spurious results due to misalignment artefacts. Putative recombinants were then realigned with their parental donors and reassessed in isolation for the predicted recombination event in RDP3 followed by a further check in GARD. The selection criteria for recombinants was set at p<0.05 by KH-testing in GARD and three or more methods with p<0.05 in the RDP suite.

### Protein homologue searches and directionality of colonization

Protein sequences of one member of a monophyletic virus group that was consistently identified in three or more ML trees were used to search for the closest homologues within picorna-like viruses. A first screen was done using the Blast algorithm with setting to exclude the input virus species. A Position-Specific Scoring Matrix (PSSM) was then generated for each protein using PSI-BLAST (position-specific iterative basic alignment search tool) with no exclusion [40]. All significant (picorna-like virus) hits were recorded. Searches were made using individual virus sequences at an inclusion threshold of 0.01. Iterations were continued for a maximum of five. The same approach was used for an ‘inverse’ PSI-BLAST search for determining the host distribution of known related sequences using the most significant (lowest e-value) animal infecting viral protein that was identified as being significantly homologous. Virus species were identified at two levels using definitions provided at the NCBI taxonomy (http://www.ncbi.nlm.nih.gov/taxonomy) and ICTV (http://ictvonline.org/virusTaxonomy.asp) databases. An e-value of 10 was used as the threshold if the hit was with a viral functional homolog.

## Results

In order to gain an understanding of the evolutionary tendencies of the *Secoviridae* family we began by dividing our analyses into the two principal components of virus evolution, 1) mutation (or associated elements: selection pressures and substitution rates) and, 2) recombination (or modularity).

### Strong negative selection pressures are evident for all species’ CPs

Prior to analysis all alignments were checked for recombination using the GARD algorithm [41] and any recombinant molecules removed from the alignment (Table S1 and File S1-File S9). The coat protein dataset was divided into two formats for analysis; full-length (ArMV, BPMV and RTSV), corresponding to discrete domain sequences with predicted cleavage product ends and shorter partial sequences (Table S2). Differences between the d_N_/d_S_ ratios were not statistically significant (overlapping confidence intervals of 0.031–0.050, 0.030–0.058 and 0.033–0.053, respectively), with evidence of strong purifying selection (0.039, 0.043, 0.042, respectively) (Table 2). For partial CP sequences d_N_/d_S_ ratios were more variable (ranging from 0.014 to 0.088), but were in-line with the full-length ratios, averaging 0.045. The total percentage of negatively selected codons varied depended on the method used, SLAC or FUBAR, the latter identifying many more. FUBAR also identified some positively selected codons where SLAC found none. These variations are attributed to the documented power and sensitivities of the methods employed [32]. In most cases the proportion of negatively selected sites detected by FUBAR was close or more than 50% (ArMV 60%, BPMV 35%, RTSV 60%, BBWV2 93%, (*Blackcurrant reversion virus*) BRV 49%, GFLV 71%, SMoV 30%, *Tomato ringspot virus* (ToRSV) 61%, *Tobacco ringspot virus* (TRSV) 15%). Lower values for BPMV, SMoV and TRSV were also found using SLAC, and are reflected in the lower diversities obtained. The shorter sequences used for SMoV and TRSV might also be a reason for these differences. In addition to a global and codon-based calculations of selection pressures, we tested the alignments for the possibility of lineage specific variations in selection pressure, and were able to find significant (p<0.05) evidence of episodic diversifying selection for two isolates of RTSV ((accession number underlined) U70989 p = 7.01×10^−6^, U71440 p = 7.93×10^−3^), and one isolate each for GFLV (EU702441 p = 6.10×10^−3^) and ToRSV (AF135414 p = 5.80×10^−5^). Removal of these isolates from the d_N_/d_S_ analyses did not significantly alter the original values obtained. Significantly, FUBAR was also able to identify positively selected codons, one and two respectively, for both GFLV and RTSV.

**Table 2:** Selection and diversity for coat protein (CP) of secovirid species.

Virus	dN/dS^c^	CI^d^	Codons	Negative Sites^e^	Positive Sites^e^	Diversity^f^	N^g^
ArMV^a^	0.039	0.031–0.050	505	34/304	0/0	0.14 (0.01)	6
BPMV	0.043	0.030–0.058	572	0/202	0/0	0.07 (0.01)	8
RTSV	0.042	0.033–0.053	706	22/422	0/2	0.10 (0.01)	10
BBWV2^b^	0.038	0.034–0.043	604	356/561	0/0	0.21 (0.01)	24
BRV	0.051	0.037–0.069	305	18/149	0/0	0.11 (0.01)	9
GFLV	0.054	0.043–0.066	489	37/349	0/1	0.11 (0.01)	13
SMoV	0.026	0.012–0.047	108	8/32	0/0	0.09 (0.01)	13
ToRSV	0.088	0.076–0.100	562	17/341	0/0	0.12 (0.01)	11
TRSV	0.014	0.002–0.043	114	2/16	0/0	0.05 (0.01)	12

*Arabis mosaic virus *(ArMV), *Bean pod mottle virus *(BPMV), *Blackcurrant reversion virus *(BRV), *Broadbean wilt virus-2 *(BBWV-2), *Grapevine fanleaf virus *(GFLV), *Rice tungro spherical virus *(RTSV), *Strawberry mottle virus *(SMoV), *Tomato ringspot virus *(ToRSV) *and Tobacco ringspot virus *(TRSV).

a– full-length CP sequences.

b– partial CP sequences.

c – ratio determined by SLAC method.

d – confidence intervals of dN/dS ratios.

e – as calculated by SLAC/FUBAR methods: p<0.05 (SLAC) posterior probability >0.9 (FUBAR).

f – standard error in parentheses.

g – number of sequences analysed.

### Coat protein nucleotide substitution rates

**Figure 2:**
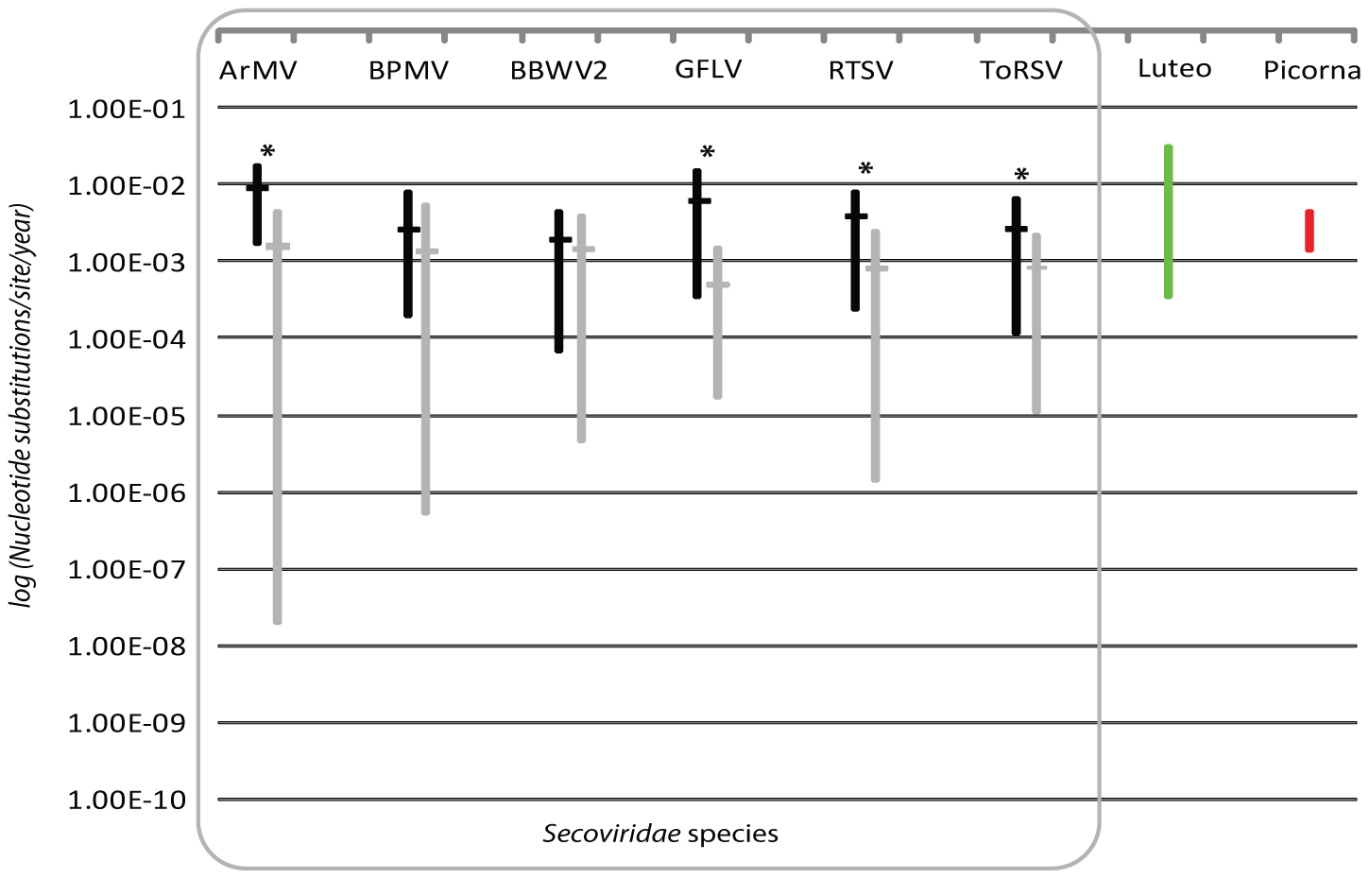
Estimates of the nucleotide substitution rates per site for the coat proteins of nine secovirids. *Arabis mosaic virus* (ArMV), *Bean pod mottle virus* (BPMV), *Broad bean wilt virus-2* (BBWV-2), *Blackcurrant reversion virus* (BRV), *Grapevine fanleaf virus* (GFLV), *Rice tungro spherical virus* (RTSV), *Strawberry mottle virus* (SMoV), *Tomato ringspot virus* (ToRSV) and *Tobacco ringspot virus* (TRSV). Real (time-tipped) mean values (black horizontal bars) are aligned with randomized (gray horizontal line) with the 95% highest posterior density (HPD) ranges for each depicted by the vertical bars. * - estimates considered statistically solid based on mean values being outside the range of the randomized HPD. Substitution rates for data spreading less than 10 years (BRV and SMoV) or where the HPD ranges was larger in the randomized than in the time-tipped calculations (TRSV) (See Table 3) are not depicted. On the right are depicted the mean substitution rate distributions for coat proteins of the *Luteoviridae* (green bar) (Pagan and Holmes 2011), and the VP1 of *Picornaviridae* (Hicks and Duffy, 2011).

The substitution rates of the CP of nine species were estimated using BEAST. We focused our attention on the CP as this domain provided the most sequences to allow for comparison and to provide an informative range of potential values. Our approach was to screen ‘time-stamped’ alignments [42] of sequences using a combination of priors and select the best model by means of Bayes Factor (BF) comparisons. Two prior models for nucleotide substitution were compared; the simpler Hasegawa, Kishino and Yano (HKY85) model and the optimal model for each alignment selected previously by JModeltest [33]. Using this approach yielded rate estimates with relatively narrow 95% HPD ranges, with the lowest value being 7.13×10^−5^ for *Broad bean wilt virus-2* (BBWV2); a value of ≤1×10^−7^ being proposed as an indicator of unreliability [43] (Table 3; Fig 2). The most favored clock and tree priors for most species was exponential with the JModeltest selected model being preferred in all cases. Estimated rates for all CPs ranged over one order of magnitude (7.22×10^−2^ – 1.21×10^−3^). However, the values for BRV and SMoV were taken to be unreliable because of the limited date range collection period (<10 years). The strength of the temporal signal in the sequences was tested by comparing ‘time-stamped’ and randomized datasets. In all cases, except TRSV, the 95% HPD range was greater in the randomized data than in the ‘time-stamped’ data; the former ranging from 3 to 5 orders of magnitude compared to 1 to 3 for the former. In six cases (ArMV, BRV, GFLV, RTSV, SMoV and ToRSV) the 95% HPD values of the randomized data fell outside the mean rates estimated for the ‘time-stamped’ data, suggesting the differences were significant and that the temporal structure was sufficient for reliable rate estimation. A temporal structure was also supported (larger R^2^ values in time-stamped trees) for the majority of species in regression analyses (Table S3). Removal or inclusion of sequences with recombinant signals from the alignments, as was the case for ArMV, BBWV2 and GFLV, did not alter these findings.

**Table 3:** Calculated Nucleotide substitution rates and Time from Most Recent Common Ancestor (TMRCA) for members of the *Secoviridae*.

Virus^a^	Date Range	#^b^	Mean Rate^c^	Mean Rate HPD^d^	TMRCA^e^	TMRCA HPD	Clock Model^f^	Tree Prior	Site Model
ArMV	1991–2008	7	**9.29E-03**	**1.86E-3 to 1.67E-2**	48.8	17.0 to 56.1	Exponential	Constant	GTR+I+G
			1.62E-03	2.04E-8 to 4.17E-3					
BBWV2	1998–2012	25	**1.99E-03**	**7.13E-5 to 4.28E-3**	225.0	40.2 to 584.0	Exponential	Exponential	TN93+G+I
			1.49E-03	4.88E-6 to 3.69E-3					
BPMV	1991–2009	8	**2.69E-03**	**2.12E-4 to 7.65E-3**	106.4	19.0 to 226.9	Lognormal	Exponential	GTR+I
			1.40E-03	5.63E-7 to 5.250E-3					
BRV	1999–2006	9	**3.43E-02**	**1.02E-2 to 5.38E-2**	10.5	9.0 to 13.2	Exponential	Constant	GTR+I
			3.30E-03	2.28E-9 to 1.02E-2					
GFLV	1991–2011	21	**7.32E-03**	**6.29E-04 to 1.27E-02**	34.5	20.0 to 63.4	Exponential	Exponential	GTR+G
			7.22E-04	3.03E-05 to 1.58E-03					
RTSV	1995–2009	10	**3.99E-03**	**2.56E-4 to 7.80E-3**	56.3	15.7 to 127.8	Exponential	Exponential	GTR+G
			8.36E-04	1.48E-6 to 2.36E-3					
SMoV	2002–2006	13	**7.22E-02**	**8.56E-3 to 0.1332**	6.3	4.1 to 12.3	Exponential	Exponential	GTR+I+G
			9.68E-03	2.37E-4 to 2.43E-2					
ToRSV	1991–2007	11	**2.74E-03**	**1.23E-4 to 6.23E-3**	154.1	18.3 to 420.9	Exponential	Exponential	HKY+I
			8.57E-04	1.30E-5 to 2.36E-3					
TRSV	1993–2010	12	**1.21E-03**	**6.55E-6 to 3.53E-3**	101.1	17.0 to 296.8	Lognormal	Exponential	GTR+I
			7.05E-04	2.00E-5 to 1.65E-3					

a - *Arabis mosaic virus *(ArMV), *Bean pod mottle virus* (BPMV), *Blackcurrant reversion virus *(BRV), *Broadbean wilt virus-2 *(BBWV-2), *Grapevine fanleaf virus* (GFLV), *Rice tungro spherical virus* (RTSV). *Strawberry mottle virus* (SMoV), *Tomato ringspot virus* (ToRSV) *and Tobacco ringspot virus* (TRSV).

b – number sequences analyzed.

c - nucleotide substitutions per site per year. Time-tipped data (in bold), randomized data (regular font).

d – HPD – highest posterior density (95%).

e - Time from most recent common ancestor.

f - Clock model, Tree prior, Site model – optimal Bayesian priors used in calculated using Bayes factors.

### TMRCAs of the *Comovirinae*

**Figure 3:**
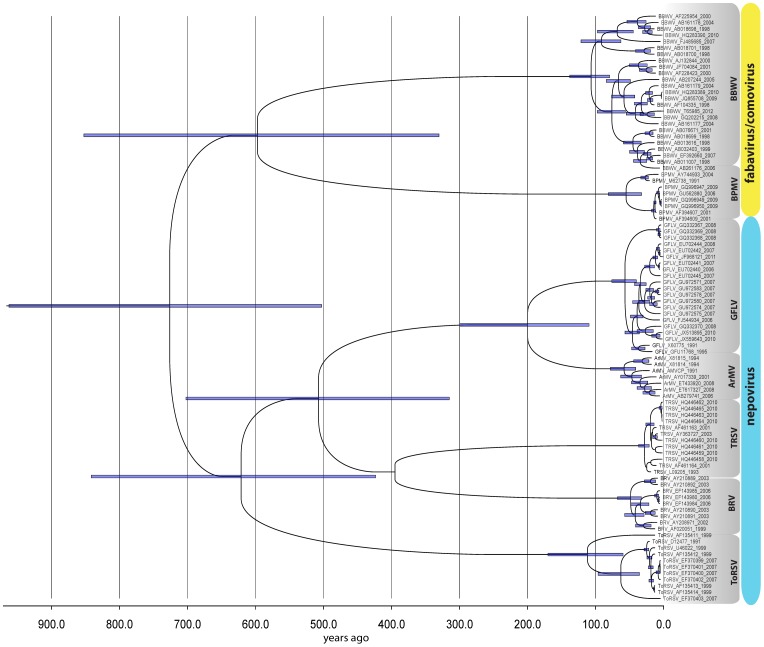
Coalescent tree for the subfamily *Comovirinae* using coat protein sequences. Bayesian priors were set for a lognormal relaxed molecular clock at the most conservative mean species nucleotide substitution estimate of 2.74×10-3 (for ToRSV) using a constant tree prior with all species’ sequences ‘time-stamped’. Horizontal blue bars show the 95% highest posterior density (HPD) ranges for each node. The presence of the HPD bar denotes a node posterior probability greater than 0.9. *Arabis mosaic virus* (ArMV), *Bean pod mottle virus* (BPMV), *Broad bean wilt virus-2* (BBWV-2), *Blackcurrant reversion virus* (BRV), *Grapevine fanleaf virus* (GFLV), *Tomato ringspot virus* (ToRSV) and *Tobacco ringspot virus* (TRSV). The equivalent tree generated using a strict clock is shown in the supplemental data.

Based on the substitution rate estimates obtained we wanted to gain an understanding of the timescale for the emergence of secovirids and their evolutionary history. To do this we employed two approaches: 1) defining the calibration nodes using the pre-determined TMRCAs for each virus species in conjunction with a Yule process (a simple model of speciation more appropriate for sequences from different species) tree prior, and 2) using the most conservative reliable mean estimate of the nucleotide substitution rate calculated for the CP data (ToRSV 2.74×10^−3^) as the rate prior in time-stamped alignments. Clock priors were varied (strict, exponential and lognormal) with the optimum combination of priors being selected using Bayes factors in the BEAST-associated program Tracer. Our initial attempts to generate a full tree for all members of the *Secoviridae* were unsuccessful due to nucleotide saturation. As a result, by focusing specifically on the *Comovirinae* subfamily, we were able to generate a coalescent tree using both a lognormal relaxed (Fig 3) and a strict clock (Fig S1), the latter having the larger marginal likelihood (Bayes factor 82.8). For the lognormal tree the root was calculated at 726 years (502–962) with the nepovirus genus TMRCA at 621 years (423–841) and the fabavirus/comovirus TMRCA at 597 years (330–852). For the strict clock tree these values were 944 (832–1063), 893 (777–1014) and 878 (751–1010), respectively. For either clock method individual species have TMRCAs close to or below 100 years; the two oldest virus TMRCA lineages being ToRSV (112 (log) and 108 (strict) years) and BBWV2 (107 (log) and 99 (strict) years), with ArMV and GFLV separating some 200 years ago.

### Detection of genetic exchange is limited

**Figure 4:**
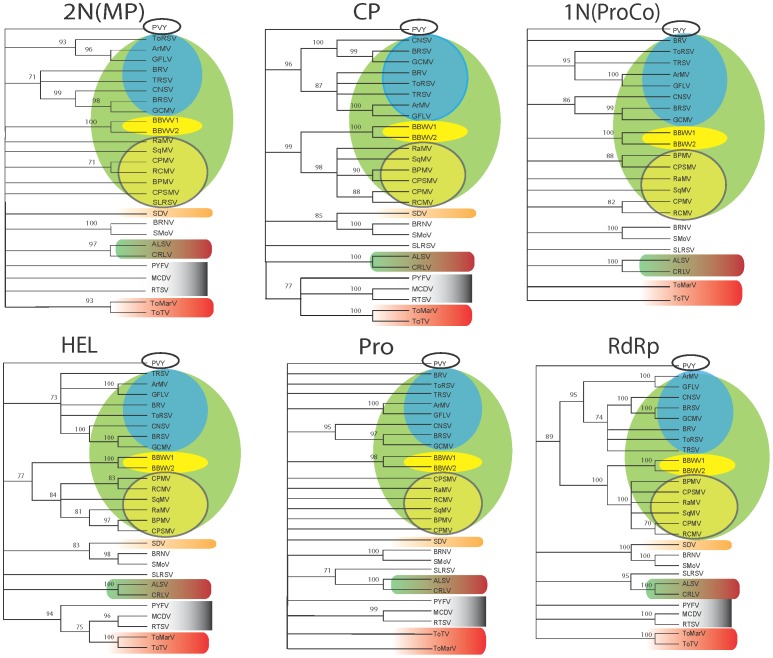
Maximum likelihood inferred phylogenetic trees for the six main functional domains of the *Secoviridae*. The putative movement protein or 2N terminal protein, 2N(MP); the coat protein (CP); the putative protease cofactor or 1N terminal protein, 1N(ProCo); the helicase (HEL), the protease (Pro), and the RNA-dependent RNA polymerase (RdRp). Designated viral lineages are contained within the polygons, in descending order: green, *Comovirinae*; blue, *Nepovirus*; yellow, *Fabavirus*; yellow/green, *Comovirus*; orange, *Sadwavirus*; red/green, *Cheravirus*; grey, *Sequivirus, Waikavirus*; red, *Torradovirus*. Numbers at nodes refer to bootstrap values, with any branch below 70% being collapsed. The outgroup (black oval) for all trees is the corresponding functional gene of the *Potato virus Y* (PVY). PVY is type member of the potyvirus genus in the *Potyviridae* family – a group of plant infecting picorna-like viruses that lies outside the Picornavirales order [74]. *Apple latent spherical virus* (ALSV), *Arabis mosaic virus* (ArMV), *Bean pod mottle virus* (BPMV), *Beet ringspot virus* (BRSV), *Black raspberry necrosis virus* (BRNV), *Blackcurrant reversion virus* (BRV), *Broad bean wilt virus-1* (BBWV1), *Broad bean wilt virus-2* (BBWV2), *Cherry rasp leaf virus* (CRLV), *Cowpea mosaic virus* (CPMV), *Cowpea severe mosaic virus* (CPSMV), *Cycas necrotic stunt virus* (CNSV), *Grapevine chrome mosaic* (GCMV), *Grapevine fanleaf virus* (GFLV), *Maize chlorotic dwarf virus* (MCDV), *Parsnip yellow fleck virus* (PYFV), *Radish mosaic virus* (RaMV), *Red clover mottle virus* (RCMV), Rice tungro spherical virus (RTSV), *Satsuma dwarf virus* (SDV), *Squash mosaic virus (*SqMV), *Strawberry mottle virus* (SMoV), *Strawberry latent ringspot virus* (SLRSV), *Tobacco ringspot virus* (TRSV), *Tomato marchitez virus* (ToMarV) and *Tomato ringspot virus* (ToRSV), *Tomato torrado virus* (ToTV).

The existence of potential chimeric-like sequences [4], [25] and reports of the evidence of recombination in secovirids in this work and others [17], [18], [23], [24] led us to look for evidence of recombination across the whole genome. Initially, we wanted to use the recombination algorithms (RDP3 and GARD) described above to screen alignments of full-length sequences, firstly using the RNA sequence so as to include the untranslated regions (UTR) and secondly, using the codons of open reading frames. Given the computational demands involved in processing and the inability of the programs used to align according to the known functional domains we opted to individually align each discrete functional domain (1N(ProCo), HEL, PRO, RdRp, 2N(MP) and CP) predicted as a post-proteolytic cleavage product. From these alignments were generated ML trees for six of the functional domains (Fig.4); the VPg was omitted from further analyses because of the failure to generate a ML tree, as were the 5’ and 3’UTR. The ML-derived cladograms shown represent the most conservative statistically significant topology for each domain. When analyzed as a whole, all alignments, except for the RdRp, showed significant saturation. Saturation was however within acceptable limits when analyzing alignments that contained only those sequences representative for each significant branch, as determined by the bootstrap values. As we were only looking for incongruences within these branches for each functional domain, saturation due to the presence of collapsed branches were deemed inconsequential. The most structured trees are those for the RdRp, HEL and CP; the *Comovirinae* are monophyletic only for the RdRp separating into two distinctively separate clades containing the nepoviruses in one and the comoviruses/fabaviruses in the other - for the HEL and CP. Notably, there is no significant topological difference between the trees that would be indicative of recombination or a modular genome makeup except for a collapse of clades to below the significance level probably due to saturation and an inability to align those sequences due to extreme divergence. Such a situation could be result of higher mutation rates, genetic drift and/or recombination. If recombination had occurred then failure to detect it could either be because of nucleotide substitution saturation in the alignment or because the hypothetical parental donor(s) is external to the *Secoviridae*. Instead, what is seen are relatively consistent similarities throughout with monophylies for the non-*Comovirinae* species for at least three domains with the ALSV/CRLV (cheraviruses), BRNV/SMoV/SDV, ToTV/ToMarV (torradoviruses) and MCDV/RTSV (waikaviruses).

Using the interspecific domain alignments described above neither GARD nor RDP3 found significant evidence of recombination. We were also unable to demonstrate any significant change in phylogenetic similarities that might be indicative of recombination by comparing ML trees. Another approach to identifying recombinants, particularly for alignments of relatively divergent viruses is to reduce noise due to potential misalignment by aligning only those sequences with higher identities [39]. To aid in this process we progressively aligned gene sequences according to their groupings in the ML trees (Fig.4) moving from larger to smaller clades and then screened them by RDP3 with a final confirmation using GARD. Here too, no significant recombination event was detected (not shown). At an intraspecific level when determining selection pressures, however, recombinants were detected for ArMV, BBWV2 and GFLV (Table S1).

### Homologies between secovirus and animal virus protein domains are significant and often unique

**Figure 5:**
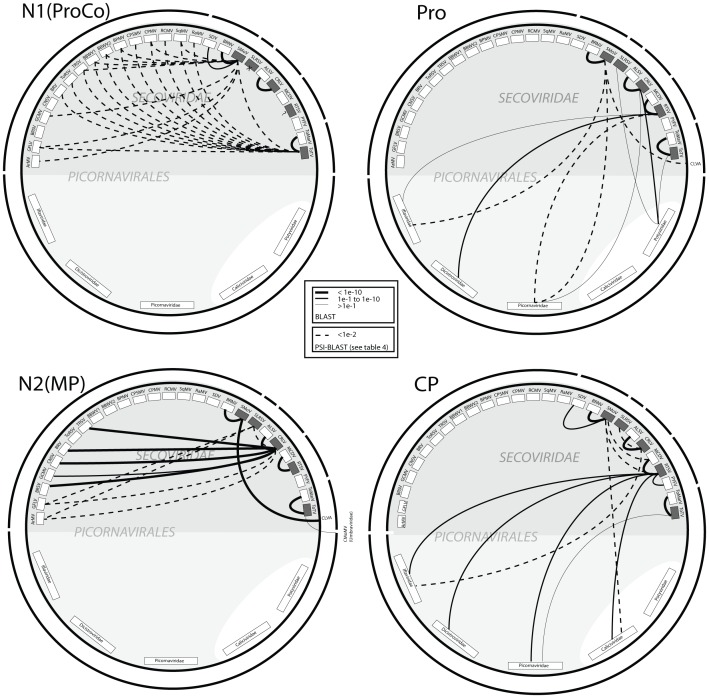
Schematics of the amino acid similarities four functional domains of members of the *Secoviridae*. These proteins are: on RNA1 (a), the putative protease cofactor or 1N terminal protein 1N(ProCo), the protease (Pro), and on RNA2 (b) the putative movement protein or 2N terminal protein 2N(MP), and the coat protein (CP). The analyzed proteins are those from five lineages of secovirids, the species *Apple latent spherical virus* (ALSV), *Arabis mosaic virus* (ArMV), *Bean pod mottle virus* (BPMV), *Beet ringspot virus* (BRSV), *Black raspberry necrosis virus* (BRNV), *Blackcurrant reversion virus* (BRV), *Broad bean wilt virus-1* (BBWV1), *Broad bean wilt virus-2* (BBWV2), *Cherry rasp leaf virus* (CRLV), *Cowpea mosaic virus* (CPMV), *Cowpea severe mosaic virus* (CPSMV), *Cycas necrotic stunt virus* (CNSV), *Grapevine chrome mosaic* (GCMV), *Grapevine fanleaf virus* (GFLV), *Maize chlorotic dwarf virus* (MCDV), *Parsnip yellow fleck virus* (PYFV), *Radish mosaic virus* (RaMV), *Red clover mottle virus* (RCMV), Rice tungro spherical virus (RTSV), *Satsuma dwarf virus* (SDV), *Squash mosaic virus (*SqMV), *Strawberry mottle virus* (SMoV), *Strawberry latent ringspot virus* (SLRSV), *Tobacco ringspot virus* (TRSV), *Tomato marchitez virus* (ToMarV) and *Tomato ringspot virus* (ToRSV), *Tomato torrado virus* (ToTV). In the lower light gray shaded hemisphere are the animal infecting families of the order Picornavirales, which excludes the picorna-like virus families Caliciviridae and Potyviridae. The outer broken black circle shows the clustering of related viruses in different secovirus lineages. Similarities were determined using the BLAST and PSI-BLAST algorithms; details of the PSI-BLAST are listed in table 4. Viruses depicted were selected based on the highest scoring sequence for each species (within the Secoviridae) or family (among the picorna-like viruses) with only one sequence hit being shown irrespective of the number identified by the program. Secovirids not included in primary analysis were only marked on the figure when they were the only representative that was a hit. For each taxon shown, Blast hits are depicted in favor of PSI-BLAST hits, PSI-BLAST hits only being shown in the absence of a Blast hit.

To further examine the possible reasons for low similarities between specific virus lineages in the ML analyses, we focused our attention on protein sequences and extended our search to include all sequences in the Genbank database. By searching for distantly related protein sequences using both the BLAST and PSI-BLAST algorithms we were able to identify putative picorna-like homologs for each functional domain. Based on the ML tree data we concentrated on non-*Comovirinae* viruses, choosing a representative virus for each apparent monophyly (eg. CRLV (representing an ALSV and CRLV clade), SMoV (representing a BRNV, SDV, SMoV clade), SLRSV alone, and ToTV (representing a ToTV, ToMarV clade) and RTSV (representing the monopartite clade of MCDV PYFV, and RTSV)) to initiate the search and generate a first Position-Specific Scoring Matrix (PSSM). Use of another representative from the same clade consistently produced similar hits to the same viruses. We principally focused on the non-replication association genome domains as these were the most divergent. The results for the 1N(ProCo), PRO, 2N(MP) and CP showed strikingly different associations for each gene (Fig 5; Table S4). For example, taking the 1N(ProCo) results, SLRSV had no significant homolog in the database, CRLV had similarity only to ALSV, and SMoV had highest homology to BRNV and ToTV, but also significant homology to a number of members of the *Comovirinae* (Fig 5; Table S4). In contrast, for the PRO domain almost all similarities detected were outside of the *Secoviridae*; SMoV having homologies to *Iflaviridae* and *Picornaviridae* proteases. CRLV PRO has significant similarity, using BLAST, with the NIa-Pro protein of the potyvirus *Keunjorong mosaic virus.* A common ancestry for the PRO domains in potyviruses is further supported by homology (BLAST) of the SLRSV PRO to the NIa-Pro of *Potato virus V*. A similar case of associations is found for the CP (Fig 5, Table S4) with no homology found with members of the *Comovirinae.* CRLV, RTSV and ToTV lineages all have high CP similarities with insect and vertebrate infecting members of the *Picornavirales*, while SMoV shows similarity to the waikavirus MCDV and two calicivirids, all of which are monopartite. Predictably, similarities for the MP are found mainly within the *Secoviridae*. CRLV and SLRSV have similarities to nepovirus MPs, SMoV is more isolated in its similarities, while ToTV surprisingly has PSI-BLAST hits with movement proteins from another plant virus family, namely, the *Umbraviridae*, re-inforcing its currently tentative assigned function as a movement protein [12]. BLAST analyses of the RdRp and HEL domains of the above virus lineages and for the three type members of the *Comovirinae* genera revealed significant interfamily similarities, along with strong (lower e-values) similarities to animal-infecting picorna-like viruses (Table S5).

### Directionality of host colonization

**Figure 6:**
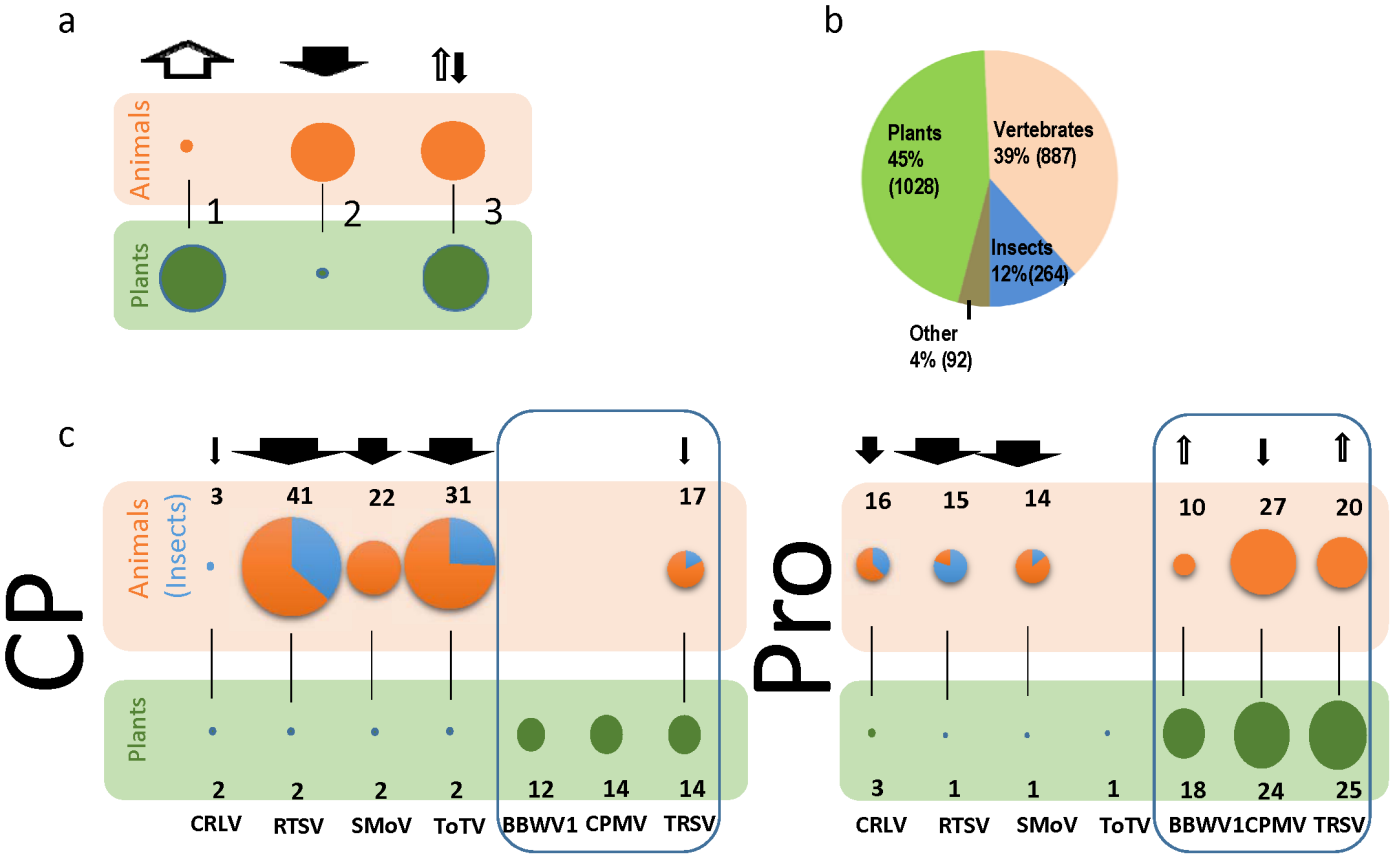
Host distribution of known related virus sequences. a) three putative models for assigning directionality of colonization based on diversity (proportional to the diameter of circle); the greater the diversity the older the population. In model three the distributions have reached an ‘equilibrium’ due to the limits of detection (ie the most divergent viruses cannot be identified), b) the distribution of the total number (in parentheses) of recognized ICTV classified viruses according to eukaryotic host. c) distribution, within the framework of the models proposed in a), of related ICTV-recognized virus species (black numbers) for the coat protein (CP, left) and the protease (Pro, right). Circles are proportional to number of species (orange – vertebrate host, blue – insect host). Arrow sizes are proportional to the ratio of the number of species in each host type (e.g. animal species:plant species or plant species:animal species). Absence of the thin black vertical line means that no significant animal homolog was identified. The rounded blue boxes contain members of the subfamily *Comovirinae*. *Broad bean wilt virus-1* (BBWV1), *Cherry rasp leaf virus* (CRLV), *Grapevine fanleaf virus* (GFLV), *Rice tungro spherical virus* (RTSV*), Strawberry mottle virus* (SMoV), *Strawberry latent ringspot virus* (SLRSV), *Tobacco ringspot virus* (TRSV), and *Tomato torrado virus* (ToTV).

The homologies identified for the CP and PRO domains is evidence of an ancestral link with animal infecting picorna-like viruses. The existence within the predominantly bipartite *Secoviridae* family of a small group of monopartite viruses (the waika- and sequiviruses) that share a higher similarity to monopartite animal-infecting viruses (Fig S2) than their bipartite relatives could suggest that viruses like RTSV are recent arrivals to the plant kingdom. The remnants of positive selection in the CP of RTSV (Table 2) supports this notion. To tentatively examine this hypothesis, we looked at the host (animal or plant) distribution of known related sequences for the CP and PRO within the simple model that the number of related sequences (per species) would be directly proportional to time within the host. (Fig 6a). Such a model requires a number of assumptions to be made (e.g. a randomly sampled population, similar rates of evolution) that could significantly alter the outcome. Therefore our interpretation should be limited to identifying a distribution that refutes the above proposed model. The results (Fig 6c and Table S6) show at least for those lineages (CRLV, RTSV, SMoV and ToTV) outside the *Comovirinae*, where the association with animal infecting picorna-like viruses is more evident, that there are no cases where the number of plant virus species’ sequences is larger than those of animal virus species’ sequences and in the majority of cases (six out of seven – no animal homolog of the ToTV PRO was identified) there are over five times as many animal virus species. For the *Comovirinae* type members (BBWV1, CPMV and TRSV) the distribution is varied, with not a single defined host population exceeding double that of the corresponding population in the other host.

## Discussion

All coat protein domains tested were found to be under extreme purifying selection, an observation already reported for members of the *Secoviridae* [14]–[16] and other plant-infecting RNA viruses [44]–[47]. It has been suggested that such extreme purifying selection is a result of selection constraints imposed by specific molecular interactions during vector transmission [47], [48]. The technique used to calculate the d_N_/d_S_ ratios itself could also possibly overestimate the degree of purifying selection by failing to detect directional selection [49]. This possibility was tested using the branch-site REL method [31] and at least for some viruses (RTSV, GFLV, ToRSV) was shown to occur, implicating episodic diversifying selection in the evolution of certain secovirid lineages, as has been recently demonstrated for a number of plant virus genes [50], [51]. In addition, analysis using the FUBAR algorithm, we were able to detect positive selection on individual codons for both RTSV and GFLV. These results therefore suggest that despite an overall strong purifying selection observed for all CPs some viruses are still undergoing restricted positive selection due to pressures to adapt to novel environments, such as new host or vector variants.

Calculations of the nucleotide substitution rates for the same species’ coat proteins obtained values ranging from 9.29×10^−03^ to 2.74×10^−03^. This compares with 1.61×10^−3^ to 5.73×10^−3^ for the VP1 of enteroviruses (*Picornaviridae*) [52], 6×10^−4^ to 3.5×10^−2^ for the CP of the *Luteoviridae* [43], and 1.17×10^−3^ for the CP of *Rice yellow mottle virus* (RYMV) [53], and falls within the general range expected for ssRNA viruses [54]. However, caution is required in interpreting these data; shorter sampling periods can potentially elevate substitution rate estimates [54], a phenomenon aptly illustrated for BRV and SMoV. The longest sampling period in this study was 20 years for GFLV, compared to 62 for enteroviruses [52], 91 for the *Luteoviridae* [43], and 40 for RYMV [53]. Therefore, although our estimates here may be correct for extant sequences the continual birth and death of lineages [49], [55] means that inferring long-term estimates is fraught with difficulties [56]. Attempts to produce a coalescent tree for the whole *Secoviridae* family proved inconsistent due to nucleotide saturation. However, by focusing only on members of the subfamily *Comovirinae,* using both lognormal relaxed and strict clock models with the most conservative substitution rate obtained at the species level (2.74×10^−3^), we were able to generate reliable (converged) trees rooted at 726 and 944 years ago, respectively. Species divergence of all extant members of the *Comovirinae* therefore is estimated to have occurred in the past 500 years. The first records of fanleaf, the oldest known grapevine virus disease, date back to 1865 [9]. ArMV, its closest relative, first reported in 1963 [9], also invariably causes fanleaf-like symptoms. Its divergence from GFLV is estimated here to have occurred around 200 years ago. Divergence estimates for the *Comovirinae* genera coincide with limited resolution at the root of both trees for the whole subfamily. Aside from the effects of heterotachy, there is also the possibility of underestimating the age of the *Comovirinae* due to the replacement of older viral lineages by younger ones [57]. Such a process – extinction and reinfection - would have to be acting across the entire phylogeny, which is less plausible for a group of viruses whose host range (wide to restricted) and type (annuals to woody perennials) is highly variable. Comparisons with other studies in plant RNA viruses using the same heretochronous sampling approach are variable in their predictions. For the *Luteoviridae* family the TMRCA was estimated at around 2,000 years ago, with the *Polerovirus* and *Luteovirus* genera TMRCAs falling around 1,000 years ago [43]. The radiation of *Potyviridae* family has been estimated around 6,600 years ago [58], while for the *Sobemovirus* genus diversification is calculated to have occurred some 3,000 years ago [53]. In our study estimates at a family level were not possible due to the limited data available. However, the genus TMRCAs determined here are not unlike those obtained for the *Luteoviridae* genera, and all extant secovirid species show a recent (>500 years) emergence in-line with the above reports and coinciding with both modern agriculture and the escalation of marine trade.

Maximum likelihood cladograms generated for each secovirid functional domain were able to identify a number of statistically unrelated lineages that pointed to a possible origin of these monophyletic groups that lay outside the *Secoviridae* family. As a result, we broadened our search using both BLAST and the PSI-BLAST algorithms; the latter looks for more distant protein homologies by pooling conserved three-dimensional protein structural information [40], [59]. The results obtained (Fig.5) clearly indicate shifting similarities of the genes analyzed, suggesting distinct evolutionary origins for each lineage. There appear to be three classes of functional domain: i) plant-ancestral domains – namely, the N-terminal proteins 1N(ProCo) and the 2N(MP) – which only have detectable similarities to other plant viruses, ii) the animal-ancestral domains – namely, the PRO and CP – which overwhelmingly have similarities with animal-infecting picorna-like viruses, and iii) the replication-associated domains – namely the HEL and RdRp – which have closer (lower e-values) similarities to other members of the *Secoviridae*, but also detectable similarities with animal-infecting picorna-like viruses. Such an arrangement produces a mosaic of viral functional domains that fits neatly into the proposed hypothesis that the Pro and CP, and the HEL and RdRp, are respective paralogous duplications of ancient proto-CP and proto-Rep genes [60]. In contrast, the plant-ancestral domains appear to represent proteins that might have been acquired while evolving in the plant. The origin of plant virus movement proteins, including those of some secovirids, is postulated to be plant-chaperon-like proteins, such as the heat-shock proteins [61].

Based on three lines of evidence, namely 1) the existence of a minority of monopartite viruses within the *Secoviridae*, 2) a higher similarity of these monopartite viruses to monopartite animal-infecting viruses, and 3) evidence of positive selection in the CP of a monopartite virus, it seems the most parsimonious hypothesis for host-switching, if we consider the *Secoviridae* as a whole, is from animal-to-plant. In order to explore this hypothesis further we used the Genbank database to analyze the distribution of related virus species according to their host (at the taxonomical rank of kingdom) on the simple assumption that the degree of speciation will be directly proportional to the age of the virus population. Clearly, there are number of caveats to this approach, including the artificial composition of the sampled population, and the possibility of different rates of evolution and extinction, and unrecognized selection pressures (for example the immune system of animals could, in theory, result in a higher selection pressure when compared to plant hosts, although the calculated selection pressures for the CP of picornaviruses [52] are as comparably low as those found in this study). To try and address the artificial composition of the sample, we examined the number of ICTV (International Committee for the Taxonomy of Viruses (http://ictvonline.org/virusTaxonomy.asp))-recognized virus species and grouped them according to their host; the assumption being that the number of animal virus species, and in particular vertebrate-infecting viruses would dominate and therefore bias any interpretation on specific distributions. Contrary to our assumption, the plant viruses made up 45% of the total number of eukaryote-infecting viruses, with vertebrate-infecting viruses and insect-infecting viruses at 39% and 12%, respectively (Fig 6b) (Table S7). The *Picornavirales* order itself reflects a similar host distribution of virus species at 53%, 27% and 16%, respectively (Table S7). Therefore the observation that at least for the CP of RTSV and ToTV and the PRO of CLRV, RTSV and SMoV there were more related virus species of insects than of plants suggested a trend supporting an ‘animal-to-plant’ colonization. For the *Comovirinae* lineage, the picture may not be so simplistic and could be either the result of an equilibrium or a more interchangeable host-switching mechanism. Another factor that could significantly alter speciation patterns is the differences in host range: if their tendency was to infect more isolated populations, secovirids could be older than related animal viruses and still have a lower prevalence than animal viruses. Although the host ranges of these viruses has not been rigorously studied there are examples of viruses with demonstrated broad (CRLV, SDV, *Cricket paralysis virus* (*Dicistroviridae*)) and assumed narrow ranges (ToTV, RTSV, *Sacbrood virus* (*Iflaviridae*), *Black queen cell virus* (*Dicistroviridae*)) [13], [62].

Outside of the plant-ancestral domains what are the possible explanations for the present similarities observed for the various functional domains? It would seem plausible that upon colonization of plants the viral RdRp and HEL genes might have evolved convergently due to high adaptive selection pressures initially imposed by interacting host cofactors that are required for virus replication [63]. Rapid convergent evolution in the replication-associated domains has been identified for a number of distinct viruses [64]–[66]. The main selection pressure on the CP would be to maintain vector transmission along with secondary roles in the infection cycle [67]. It is tempting to see the often unique similarities between secovirid CPs and animal-infecting picorna-like viruses as evidence of an older co-evolutionary relationship where present-day vectors might have been past hosts [68], [69] (Fig. S3).

**Figure 7:**
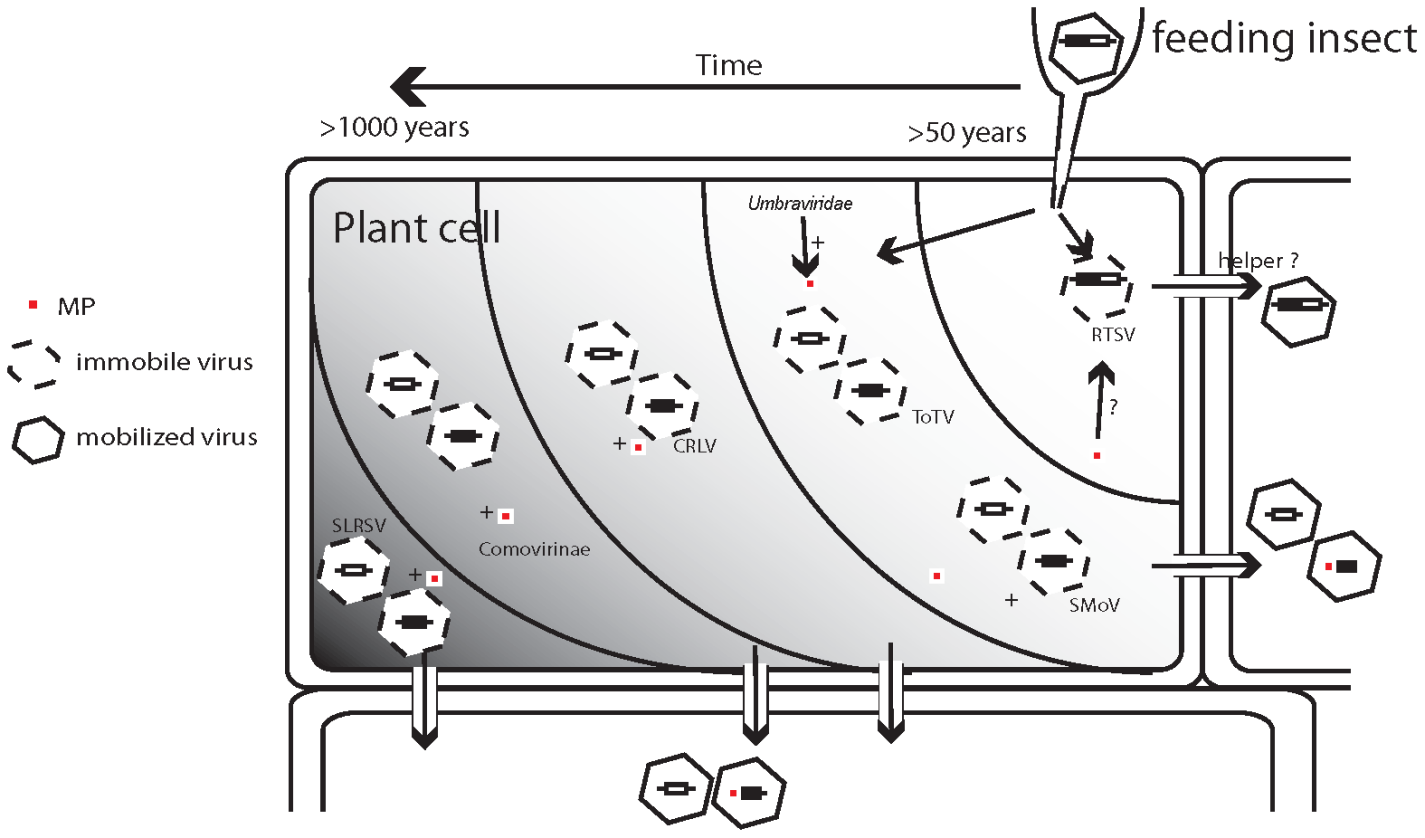
Putative model of colonization of plants by the Secoviridae. Cherry rasp leaf virus (CRLV), Rice tungro spherical virus (RTSV), Strawberry mottle virus (SMoV), Strawberry latent ringspot virus (SLRSV), Tomato torrado virus (ToTV). Time scale of years increasing from right to left. Upon first introduction from vectors into plants, the viruses can replicate, but are not able to move from cell-to-cell (schematically shown as polygons with dashed lines). Upon acquisition of a movement protein (MP, shown as red square), viruses (schematically shown as polygons with solid lines) are then more effectively able to move cell-to-cell and systemically infect the plant host.

A unidirectionality to novel host colonization may be too simplistic, but the very tight relationship between plant viruses and their vectors and the presence of related viruses in ecdysozoans presents a plausible scenario wherein ecdysozoan-infecting viruses could have been introduced during feeding into plants (Fig. 7). In this case, whether animal-to-plants or plants-to-animals, the overall process of host-switching appears to have led to a level of specialization where, in the case of secovirids, the virus appears to have lost its ability to replicate in the vector: unlike in other virus families (for example *Bunyaviridae*, *Rhabdoviridae*, and *Reoviridae*), there is no evidence of virus replication in the vector, although some nepoviruses and comoviruses are described as being persistently transmitted [70]. Recently, TRSV has been detected in honeybees and suggested to be replicating in a variety of different tissues from brain to leg. If this report can be more fully substantiated, the propagation of TRSV in an insect would provide an example of transkingdom host-switching [71]. The insect infecting *Rhopalosiphum padi virus* (*Dicistroviridae*, genus *Cripavirus*) is transmitted horizontally via plants in which it is able spread to all parts without replication [72], [73]. In theory, adaptation to a plant environment would eventually require acquisition of a movement protein (and perhaps a 1N(ProCo)) which in the case of the five secovirid lineages analyzed here appear to have occurred from a number of different sources, including from other virus families. Movement of the non-enveloped insect infecting *Flock house virus* (FHV, family *Nodaviridae*, also a picorna-like virus) [74] in the plant host *Nicotiana benthamiana* was complemented by the movement proteins of two unrelated plant viruses. Furthermore, FHV was shown to replicate in the inoculated leaves of six monocot and dicot species [75].

The only salient theoretical example of ‘plant-to-animal’ host-switching also involves a calicivirus; where it was postulated that nanoviruses, which are plant single-stranded, circular DNA viruses must have switched to an animal host and then recombined in the C-terminal of the Rep protein with the RNA of a calicivirus prior to returning to plants [76]. Such a scenario was proposed purely on the basis that all caliciviruses described so far infect invertebrates. Conceptually, the loss of the movement protein would be a more parsimonious process, but the *de novo* inoculation and colonization of insect cells by a plant virus is equally as difficult to imagine. Picornaviruses require cell-surface receptors that are involved in cell attachment, signaling and endocytosis, and in triggering of capsid structural alterations that are required for infectious entry [77].

Taken together, the results provide evidence for the recent evolutionary tendencies and putative ancestral origins of members of the *Secoviridae*, and provide the basis for further studies into these and related viruses and their ability to adapt to a huge variety of different hosts. Unanswered questions raised in the discussion here that could be addressed in future studies are: 1) Does an RdRp domain evolve rapidly within a founder population colonizing a markedly different host? 2) Is secovirid genome segmentation an unavoidable consequence of plant colonization, and under what conditions does it become selectively detrimental? 3) Are there ecdysozoan vectors harboring viruses that more closely resemble secovirids? 4) Are there emergent secovirid-like viruses that could threaten future agricultural production? 5) What are the characteristics of picorna-like viruses that favor host-switching? In answering these questions we hope to better predict the sources of emergent viruses and how to control them.

## Supporting Information

Figure S1 Comovirinae strict tree.
(TIF)

Figure S2 protein alignments.
(TIF)

Figure S3 Insect virus trees.
(TIFF)

Table S1 Recombination check
(XLSX)

Table S2 Secovirus CP Accessions.
(XLSX)

Table S3 R-squared values Time-stamped vs. Random.
(XLSX)

Table S4 PSI-BLAST.
(DOCX)

Table S5 HEL RdRp BLASTs.
(XLSX)

Table S6 Virus ICTV Species BLAST.
(XLSX)

Table S7 ICTV recognized species.
(XLS)

File S1 CP alignment dNdS.
(MSF)

File S2 (MSF)

File S3 (MSF)

File S4 (MSF)

File S5 (MSF)

File S6 (MSF)

File S7 (MSF)

File S8 (MSF)

File S9 (MSF)
