# Correction: An Evolutionary Analysis of the *Secoviridae* Family of Viruses

**DOI:** 10.1371/journal.pone.0119267

**Published:** 2015-03-18

**Authors:** The PLOS ONE Staff

There are formatting errors in Table 1, “List of Secoviridae isolates and related sequences used in the analyses, including their acronyms and Genbank accession numbers.” Please see the corrected Table 1 here.

**Table 1:** List of Secoviridae isolates and related sequences used in the analyses, including their acronyms and Genbank accession numbers.

Genome	Subfamily	Genus	Species	Acronym	RNA 1	RNA 2
Bipartite	*Comovirinae*	*Comovirus*	*Bean pod mottle virus*	BPMV	NC_003496.1	NC_003495.1
			*Cowpea mosaic virus*	CPMV	NC_003549.1	NC_003550.1
			*Cowpea severe mosaic virus*	CPSMV	NC_003545.1	NC_003544.1
			*Radish mosaic virus*	RaMV	NC_010709.1	NC_010710.1
			*Red clover mottle virus*	RCMV	NC_003741.1	NC_003738.1
			*Squash mosaic virus*	SqMV	NC_003799.1	NC_003800.1
		*Fabavirus*	*Broad bean wilt virus-1*	BBWV1	NC_005289.1	NC_005290.1
			*Broad bean wilt virus-2*	BBWV2	NC_003003.1	NC_003004.1
		*Nepovirus*	*Arabis mosaic virus*	ArMV	AY303786.1	NC_006056.1
			*Beet ringspot virus*	BRSV	NC_003693.1	NC_003694.1
			*Blackcurrant reversion virus*	BRV	NC_003509.1	NC_003502.1
			*Cycas necrotic stunt virus*	CNSV	NC_003791.1	NC_003792.2
			*Grapevine chrome mosaic*	GCMV	NC_003622.1	NC_003621.1
			*Grapevine fanleaf virus*	GFLV	NC_003615.1	NC_003623.1
			*Tomato ringspot virus*	ToRSV	NC_003840.1	NC_003839.2
			*Tobacco ringspot virus*	TRSV	NC_005097.1	NC_005096.1
	*unassigned*	*Cheravirus*	*Apple latent spherical*	ALSV	NC_003787.1	NC_003788.1
			*Cherry rasp leaf virus*	CRLV	AY764390.2	AY122330.2
		*Sadwavirus*	*Satsuma dwarf virus*	SDV	NC_003785.2	NC_003786.2
		*Torradovirus*	*Tomato marchitez virus*	ToMarV	NC_010987.1	NC_010988.1
			*Tomato torrado virus*	ToTV	DQ388879.1	DQ388880.1
		*unassigned*	*Strawberry latent ringspot virus*	SLRSV	NC_006964.1	NC_006965.1
			*Black raspberry necrosis virus*	BRNV	DQ344639.1	DQ344640.1
			*Strawberry mottle virus*	SMoV	NC_003445.1	NC_003446.1
Monopartite		*Waikavirus*	*Maize chlorotic dwarf virus*	MCDV	NC_003626.1	NC_003626.1
			*Rice tungro spherical virus*	RTSV	NC_001632.1	NC_001632.1
		*Sequivirus*	*Parsnip yellow fleck virus*	PYFV	NC_003628.1	NC_003628.1

There is an error in Fig. 5, “Schematics of the amino acid similarities four functional domains of members of the *Secoviridae*”. Please see the corrected Fig. 5 here.

**Fig 5:**
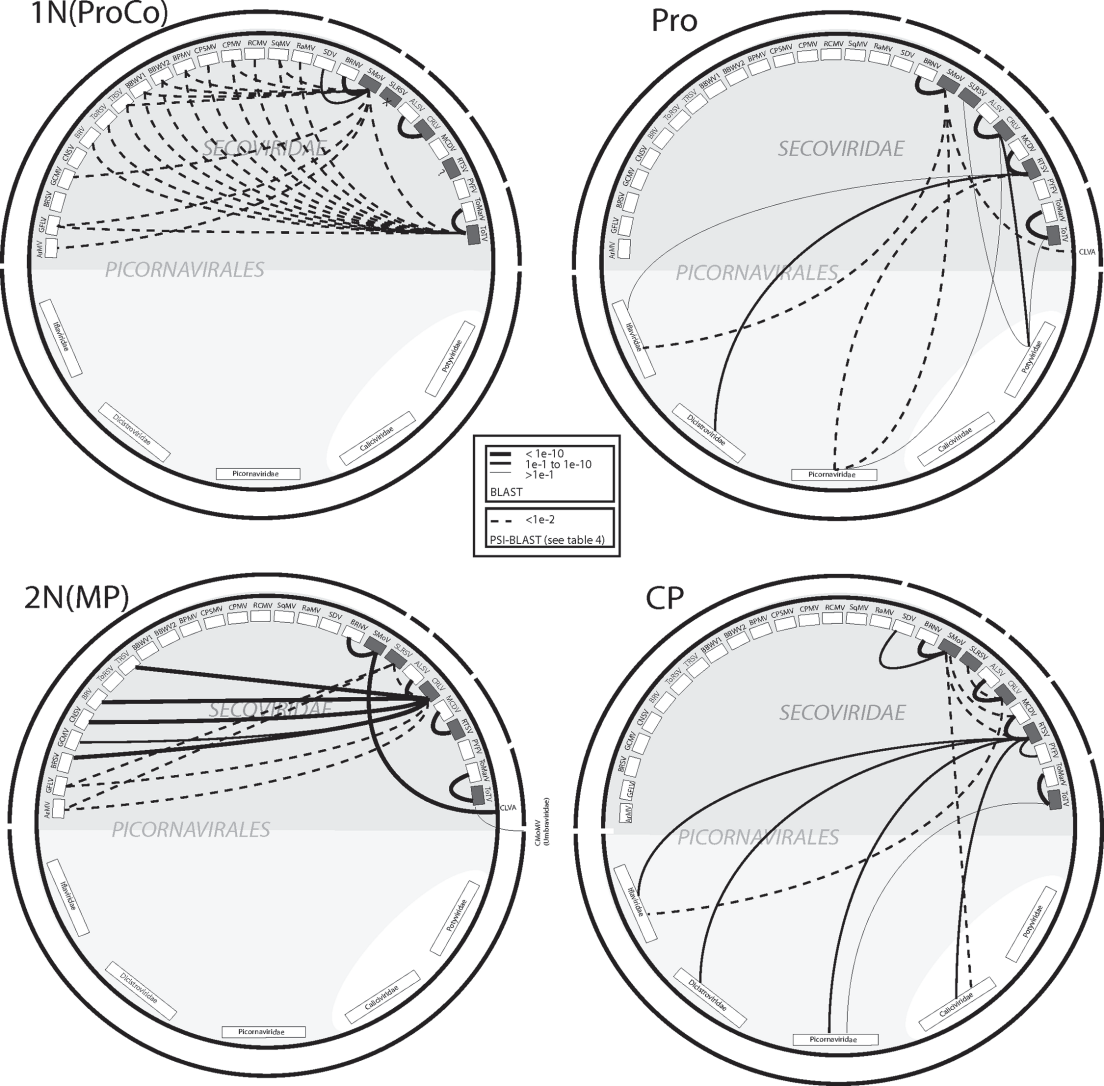
Schematics of the amino acid similarities four functional domains of members of the *Secoviridae*. These proteins are: on RNA1 (a), the putative protease cofactor or 1N terminal protein 1N(ProCo), the protease (Pro), and on RNA2 (b) the putative movement protein or 2N terminal protein 2N(MP), and the coat protein (CP). The analyzed proteins are those from five lineages of secovirids, the species *Apple latent spherical virus* (ALSV), *Arabis mosaic virus* (ArMV), *Bean pod mottle virus* (BPMV), *Beet ringspot virus* (BRSV), *Black raspberry necrosis virus* (BRNV), *Blackcurrant reversion virus* (BRV), *Broad bean wilt virus-1* (BBWV1), *Broad bean wilt virus-2* (BBWV2), *Cherry rasp leaf virus* (CRLV),*Cowpea mosaic virus* (CPMV), *Cowpea severe mosaic virus* (CPSMV), *Cycas necrotic stunt virus* (CNSV), *Grapevine chrome mosaic* (GCMV), *Grapevine fanleaf virus* (GFLV),*Maize chlorotic dwarf virus* (MCDV), *Parsnip yellow fleck virus* (PYFV), *Radish mosaic virus* (RaMV), *Red clover mottle virus* (RCMV), Rice tungro spherical virus (RTSV),*Satsuma dwarf virus* (SDV), *Squash mosaic virus* (SqMV), *Strawberry mottle virus*(SMoV), *Strawberry latent ringspot virus* (SLRSV), *Tobacco ringspot virus* (TRSV),*Tomato marchitez virus* (ToMarV) and *Tomato ringspot virus* (ToRSV), *Tomato torrado virus* (ToTV). In the lower light gray shaded hemisphere are the animal infecting families of the order Picornavirales, which excludes the picorna-like virus families Caliciviridae and Potyviridae. The outer broken black circle shows the clustering of related viruses in different secovirus lineages. Similarities were determined using the BLAST and PSI-BLAST algorithms; details of the PSI-BLAST are listed in table 4. Viruses depicted were selected based on the highest scoring sequence for each species (within the Secoviridae) or family (among the picorna-like viruses) with only one sequence hit being shown irrespective of the number identified by the program. Secovirids not included in primary analysis were only marked on the figure when they were the only representative that was a hit. For each taxon shown, Blast hits are depicted in favor of PSI-BLAST hits, PSI-BLAST hits only being shown in the absence of a Blast hit.
