# Supplementary figures and images for: An Evolutionary Analysis of the Secoviridae Family of Viruses
Source: PLoS One. 2014 Sep 2;9(9):e106305. doi: 10.1371/journal.pone.0106305 (PMC4152289; doi:10.1371/journal.pone.0106305)

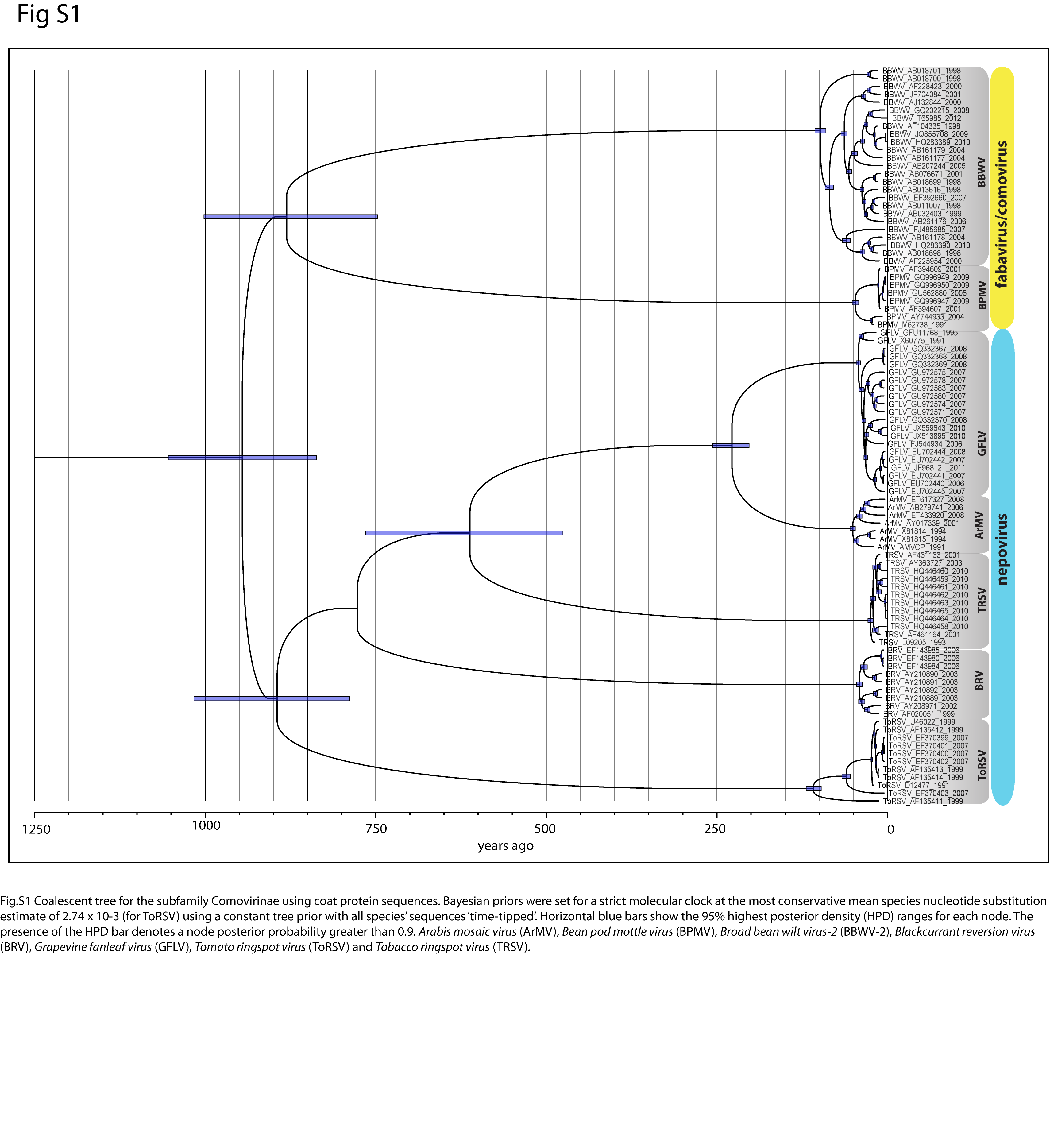

Supplement: Figure S1 — Comovirinae strict tree. (TIF) [file pone.0106305.s001.tif]

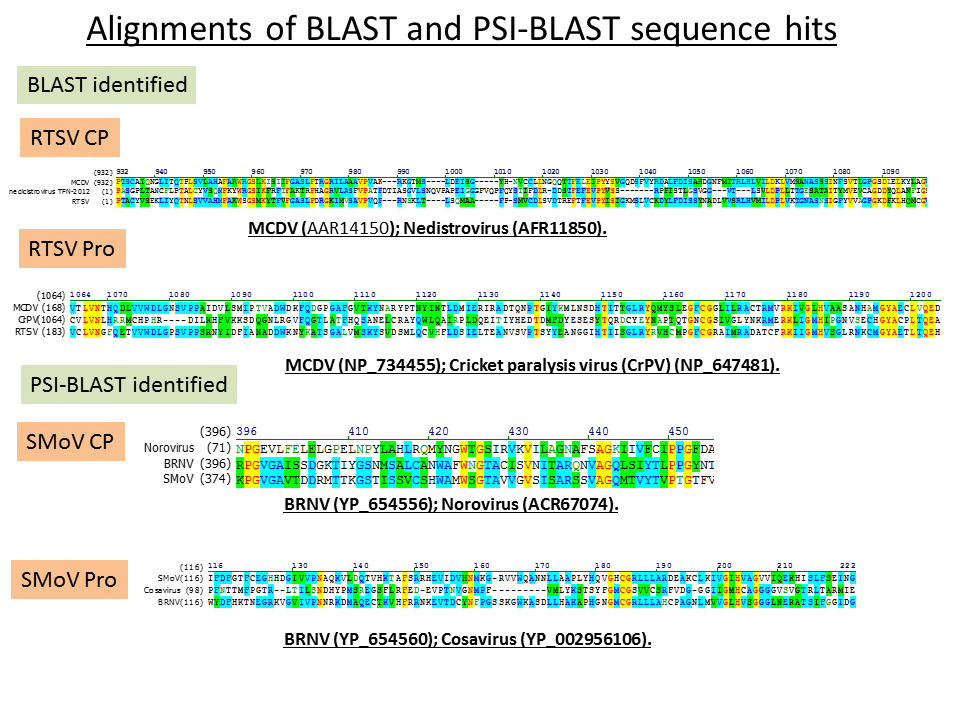

Supplement: Figure S2 — protein alignments. (TIF) [file pone.0106305.s002.tif]

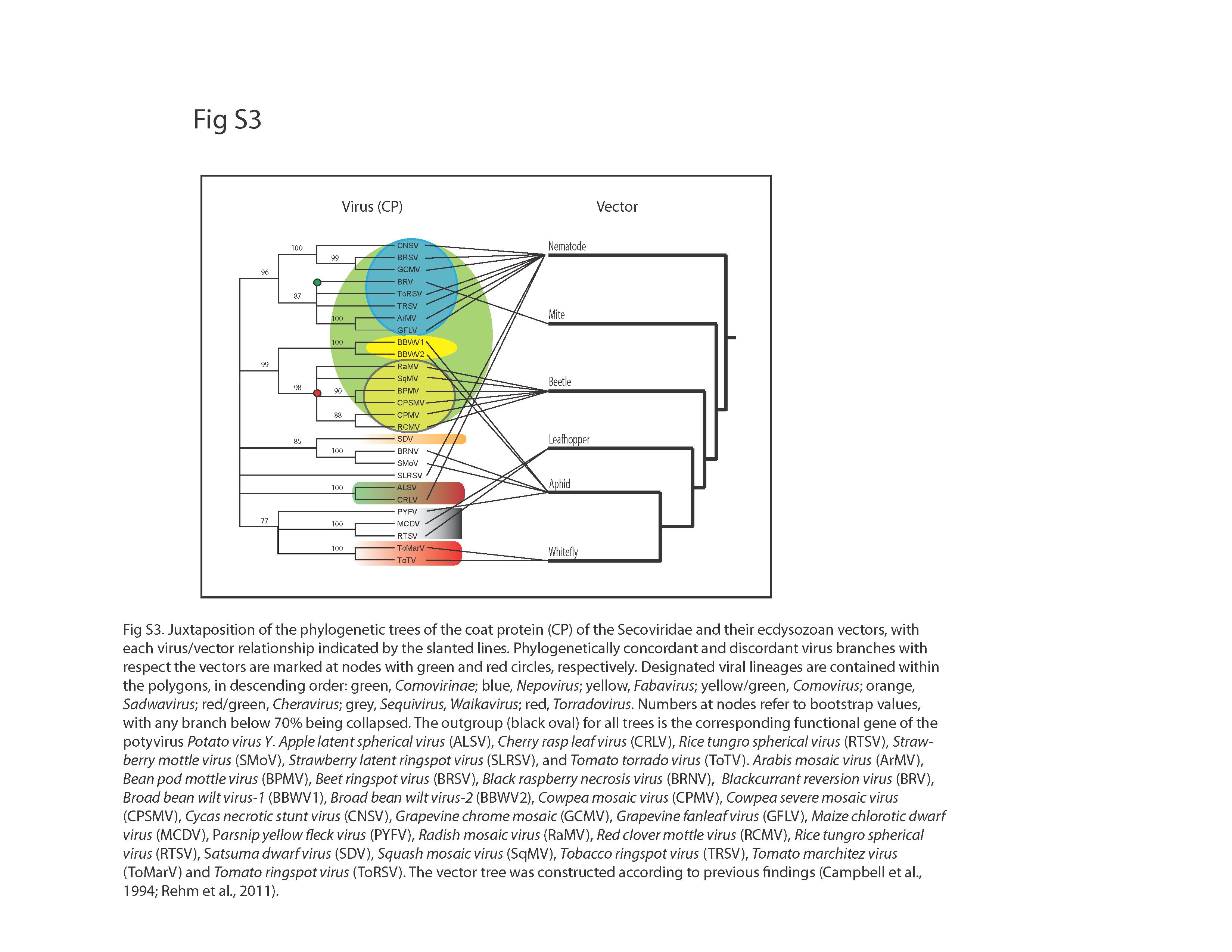

Supplement: Figure S3 — Insect virus trees. (TIFF) [file pone.0106305.s003.tiff]
