# Supplementary material for: An Evolutionary Analysis of the Secoviridae Family of Viruses
Source: PLoS One. 2014 Sep 2;9(9):e106305. doi: 10.1371/journal.pone.0106305 (PMC4152289; doi:10.1371/journal.pone.0106305)
Supplement: Table S4 — PSI-BLAST. (DOCX) [file pone.0106305.s007.docx]

Table S4. PSI-BLAST results of five lineages of Secoviridae for the 1N(ProCo), 2N(MP), CP and Pro functional domains.

Note that the table represents only those virus sequences uniquely found using PSI-BLAST: repeat hits already identified by BLAST (Fig. 5) are not included. * top five potyviruses included only. # 1N(ProCo) functional domain not identified. The CP of RTSV was not tested using PSI-BLAST due to the high numbers of hits which were sufficiently represented by the BLAST (Fig.5) results.
